# The Effect of Health Promoting Intervention on Healthy Lifestyle and Social Support in Elders: A Clinical Trial Study

**DOI:** 10.5812/ircmj.18399

**Published:** 2014-08-05

**Authors:** Abbas Rahimi Foroushani, Fatemeh Estebsari, Davoud Mostafaei, Hasan Eftekhar Ardebili, Dvoud Shojaeizadeh, Maryam Dastoorpour, Ensiyeh Jamshidi, Mohammad Hossein Taghdisi

**Affiliations:** 1 Department of Epidemiology and Biostatistics, School of Public Health, Tehran University of Medical Sciences, Tehran, IR Iran; 2 Department of Health Education and Promotion, School of Public Health, Iran University of Medical Sciences, Tehran, IR Iran; 3 Department of Health Economic and Management, School of Public Health, Tehran University of Medical Sciences, Tehran, IR Iran; 4 Department of Health Education and Promotion, School of Public Health, Tehran University of Medical Sciences, Tehran, IR Iran; 5 Modeling In Health Research Center, Futures Studies in Health Institute, Kerman University of Medical Sciences, Kerman, IR Iran; 6 Community Based Participatory Research Center, Iranian Institute for Reduction of High-risk Behaviors, Tehran University of Medical Sciences, Tehran, IR Iran

**Keywords:** Lifestyle, Social support, Health Promotion

## Abstract

**Background:**

Many of the problems pertaining to old age originate from unhealthy lifestyle and low social support. Overcoming these problems requires precise and proper policy-making and planning.

**Objectives:**

The aim of the current research is to investigate the effect of health promoting interventions on healthy lifestyle and social support in elders.

**Patients and Methods:**

This study was conducted as a clinical trial lasting for 12 months on 464 elders aged above 60 years who were under the aegis of health homes in Tehran, Iran. Participants were selected through double stage cluster sampling and then divided into intervention and control groups (232 individuals in each). Tools for gathering data were a demographic checklist and two standard questionnaires called Health-Promoting Lifestyle Profile version 2 and personal resource questionnaire part 2. Data were analyzed using descriptive and analytical tests including paired t test, analysis of covariance (ANCOVA) and Pearson correlation coefficient.

**Results:**

The average age of elders in this study was 65.9 ± 3.6 years (ranging between 60 and 73 years old). Results showed that the differences between the mean post-test scores of healthy lifestyle and its six dimensions as well as perceived social support and its five dimensions in the control and intervention groups were statistically significant (P value < 0.0001).

**Conclusions:**

Aging is an inevitable stage of life. However, effective health promoting interventions can procrastinate it, reduce its consequences and problems, and turn it into a pleasant and enjoyable part of life.

## 1. Background

Improvement of health standards has enabled many to experience aging and thus, world population is getting old. According to the prediction of United Nations Population Division, the population aged above 60 in the world grows from 800 million individuals at present to over 2 billion individuals in 2050. Also, United Nations has predicted that Iranian population aged above 60 years old will make 33 percent of the country's overall population from 2011 to 2050 with a 26% growth (1). Aging of population is a menace for the appearance of a serious challenge related to the rise of non-contagious diseases and a huge increase in care and treatment expenses (2). Some factors can procrastinate old age or reduce the intensity of changes in body among which one of the most important ones is healthy lifestyle (3).

Healthy lifestyle is known to be a major factor in preventing and controlling non-contagious diseases. Healthy lifestyle means changing unhealthy habits and following healthy behaviors and habits (4, 5). Studies conducted by World Health Organization show that about 60 percent of quality of life (6, 7) and 53 percent of causes of deaths are related to lifestyle and health behaviors (8). Moreover, living a healthy lifestyle actualizes through social support (9, 10). Social support refers to care, affection, esteem, consolation, and assistance that other individuals or groups provide for the individual (1).

Social support in elders is defined as the amount of affection, companionship, and care that family members, friends, and other individuals provide for the elder (11, 12). Research has stated that social support is related to social isolation, stress-making factors, mental disturbances, depression symptoms, amount of social interactions, and eventually, lifestyle (13). It is also considered as one of the effective factors in maintenance, continuity, and promotion of healthy lifestyle interventions (14). Given the increase in elderly population and their support, social, rehabilitation, and health-treating issues and problems have also increased. The elder's health now is a crucial and essential issue in most societies and overcoming the problems of this vulnerable group requires precise and proper policy-making and planning. Therefore, the current research investigates the effect of health promoting intervention on healthy lifestyle and social support in elders. The underlying hypothesis of this research is that health promoting interventions lead to the increase in the score average of perceived social support and change the elder's healthy lifestyle.

## 2. Objectives

The aim of the current research is to investigate the effect of health promoting interventions on healthy lifestyle and social support in elders.

## 3. Patients and Methods

### 3.1. Study Population and Sampling

Once the approval was gained from Ethics Committee of Tehran University of Medical Sciences (ethical code: 19230) and required arrangements were done, this clinical trial study (parallel) was conducted for one year on 464 elders aged above 60 who visited health homes in 22 districts of Tehran's municipality in 2013. Participants were selected through double stage cluster sampling and then divided into intervention and control groups (232 individuals in each).

The number of samples was determined based on the matrix of correlation coefficients (alpha = 0.05 and beta = 0.2). The aforementioned samples, with regard to the time and cost, were multiplied by research efficacy coefficient 1.2 and the probability of loss by 15% and finally number of samples in every group was calculated 232 in each group.

Among all health homes, 44 were selected. Then, their names were written on 44 cards. These 44 cards were scrambled and each time one card was picked out. Eventually, 22 cards were picked out. Using random method between 1 and 2, then, intervention group or control group were attributed to these 22 cards. The other 22 were allocated to the second group. In this way, based on the list of 22 administrative districts in Tehran, two health homes, one for the individual in intervention group and the other for the one in control group, were randomly selected from each district. Then, from the list of individuals in every Health Home 10 or 11 individuals (five female and six male or vice versa) who met the inclusion criteria were randomly selected. Totally, 232 in intervention group and 232 in control group entered the study.

Intervention and control groups had no contact with each other. To avoid bias, Tehran municipality's health homes were asked not to allow any other similar intervention (to one in this study) to be conducted during the time this research was carried out. Although after completing the study, all control groups also received similar education. Individuals were required to be above 60 years old, speak Persian, lack any mental and psychological disorders, and normal orientation to space and time to enter this study. The elders who lacked these criteria were not allowed to take part. To avoid confounding factors such as age, gender, marital status, level of education, etc. their effects were controlled using statistical models. Intervention and control groups were not significantly different in the baseline.

### 3.2. Data Collection Tools

#### 3.2.1. Tools for Gathering Data Included

##### 3.2.1.1. Demographics Checklist

Demographics checklists included age, sex (female, male), marital status (single, married, widowed/divorcee), level of education (elementary, middle, and higher), house ownership (tenant, landlord), experience of any chronic disease (yes, no), being insured (yes, no), life structure (by asking "with whom do you live now?" and responses of family members (spouse and children), relatives and acquaintances (sister, brother, friend, others and alone) and financial status (stable, unstable). Stable financial status was evaluated based on the retirement salary, receiving funds from other organizations and being employed while unstable financial status was marked with receiving no retirement salary or funds from other organizations and dependence upon children. Perceived health status was achieved by asking, "How do you evaluate your health conditions at the moment?" (good, average, bad) (15).

##### 3.2.1.2. Healthy Lifestyle Questionnaire

We used the questionnaire of Health-Promoting Lifestyle Profile version two (HPLP2), which included 52 questions (15). Lifestyle was measured in six dimensions: physical activity, nutrition, interpersonal relations, stress management, spiritual growth and health responsibility. Questions were designed on a Likert scale ranging from never (1) to routinely (4). Higher scores show more health-promoting behaviors. This tool has been translated into different languages (12) including Persian (16) and its validity and reliability have been confirmed (Cronbach's alpha = 0.84).

##### 3.2.1.3. Social Support Questionnaire

**Figure 1.**
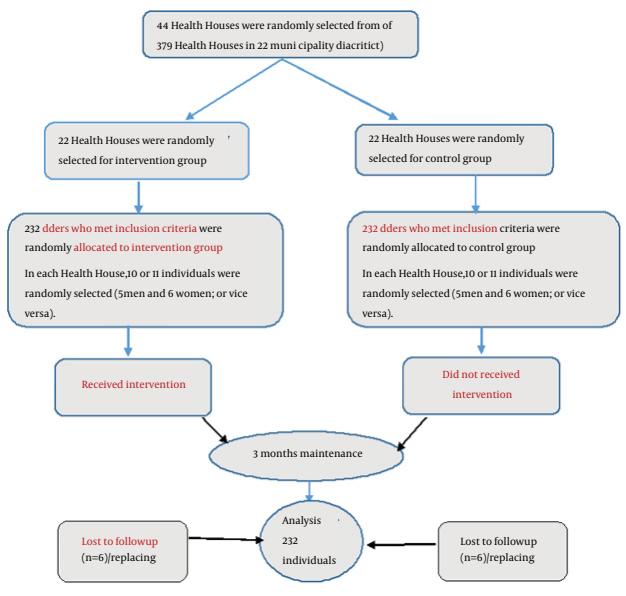
Flow Chart of RCT

The 25-item personal resource questionnaire part two (PRQ85-part 2) perceived social support of elders was utilized, measured in five dimensions: intimacy, assistance, social integration, affirmation of worth, and nurturance on a seven-option Likert scale (from strongly disagree = 1 to strongly agree = 7). Scores ranged from 25 to 175. Higher scores indicated more perceived social support and lower scores revealed less perceived social support. This questionnaire has been translated into different languages including Persian and its validity and reliability have been confirmed (Cronbach's alpha = 0.81) (9). The questionnaires in this study were anonymous and were detectable based on their specific codes. They were filled out by trained researchers in face to face interviews. Questionnaires in all intervention and control groups were filled out simultaneously (Figure 1).

### 3.3. Health Promoting Intervention

Once the initial information were gathered from intervention and control groups and initial data were analyzed, health-promoting intervention was designed with the supervision of professional health promoters and educators, gerontologists according to scientifically reliable sources. Health-promoting intervention was designed based on the areas emphasized by healthy lifestyle and social support. The booklet guide to healthy lifestyle in old age, published by ministry of health and medical education of Islamic republic of Iran, and principles of healthy old age (5), were used as our two references. Health-promoting interventions included healthy lifestyle, proper nutrition, elders’ physical activities and interpersonal relations, control of stress, night sleep, memory empowerment, and acceptance of aging (Table 1). The interventions were educated in a weekly manner in health homes of every district for two month (8 sessions each, lasting for 45 minutes).

Furthermore, educational packages and pamphlets were prepared and distributed among elders. Also, weight and blood pressure of participants in educational classes were controlled every month (as a reward for taking part in the study). Once the educational intervention was done, all contents concerning healthy lifestyle in old age educational intervention were compiled in a CD and a booklet, and were distributed free of charge among elders and their families. Moreover, health messages regarding the issue under study were designed and texted for elders on a daily basis (Table 2). Educational methods used for educating elders included lecture, questions and answers and problem solving techniques. None of these educations were provided for control group. Three months after the education was done, questionnaires were filled out by two groups once again. The information gathered in the second phase was compared to the previous ones. Once the research was done and data were gathered, the control group was studied like the intervention group.

**Table 1.** T types of Health Promoting Interventions Were Performed in This Study

Intervention context	Intervention Type
**Sleep interventions**	Allocating specific hours for sleeping, scheduling daily walks, light dinner, not drinking tea or coffee after meals, drinking a glass of warm milk, taking a hot shower before going to sleep, not taking naps during day, not consuming sleeping drugs without consulting the physician
**Physical Activity Interventions**	Recommending regular and suitable exercises, keeping proper weight, daily exercises and mobility depending on bodily conditions, slow walking in fresh air in mornings and afternoons twice each one lasting 10 to 15 minutes
**Nutritional Interventions**	Nutritional suggestions such as importance of proper nutrition in old age, eating breakfasts regularly, control and maintenance of weight, having fruits and vegetables in daily diet, consuming low-fat foods, not using much salt
**Interpersonal Relations Interventions**	Communicating with family, friends, acquaintances, and old colleagues, avoiding isolation, holding regular gatherings with friends at home or outside like a park, coffee shop, or restaurant, listening to other people's talks and pains, establishing friendly contacts with other elders and spending time with them
**Health Responsibility Interventions**	Paying regular visits to physician to prevent diseases and even diagnose them early, maintaining a close contact with health experts and asking for consultation and help if any problems arise
**Spiritual growth Interventions**	Attending religious gatherings and events, praying and paying attention to spiritual growth and development
**Stress control Interventions**	Asking others for help if necessary, involving in favorite activities and programs, spending time with friends, relatives, and other elders, going out of house, going to parks, going to shops, taking light daily walks, participating in social and group activities, helping with household chores and avoiding seclusion, having programs for life, physical activity, meditation, and progressive muscle relaxation
**Memory improvement interventions**	Doing crossword puzzles, keeping notes of important events, recounting memories, looking at photo albums, reading books and newspapers, listening to news on radio or TV, doing mental games (chess), learning new things (a new language), memorizing new things (poems), reducing the consumption of sugar, fat, and salt, eating fresh fruits and vegetables, not self-prescribing drugs, using sufficient natural resources or vitamin B_1_ and B_12_ supplements to improve memory and concentration power
**Social Support interventions**	Volunteering for social activities, registering in a group or club, doing charities, taking part in group sports and walks
**Old age acceptance**	Realizing and accepting the reality of being old, optimism toward future, not being afraid of retirement, noticing beauties of being old, engaging in doing a job or activity like growing plants, keeping pets or having an aquarium

**Table 2.** Instances of Some Health Messages Which Were Texted to Elders

Elders need fewer calories than others but they need the same amount of nutritionals
Dear elder, old age and deteriorating physical abilities are not obstacles for physical exercises
Using sufficient natural resources or vitamin B_1_ and B_12_ supplements improve memory and concentration power and effect mental health in elders
Immobility is the most important cause for early old age
Healthy old age is dependent on healthy lifestyle
Old age and deteriorating physical health are not obstacles for doing sports
Healthy lifestyle in old age reduces the risk of being effected by many diseases
Healthy lifestyle makes old age favorable, enjoyable, and healthy

### 3.4. Ethical Consideration

The participants were ensured that the data would remain confidential and used for the research purposes only. The participants were also given an unconditional and absolute right of withdrawal at any time.

### 3.5. Data Analysis

To actualize aims and hypothesis of the research and answer the questions it raised, first, the normality of data was approved by using Kolmogorov-Smirnov test. Descriptive statistics including frequency, distribution, mean and standard deviation and analytical statistical tests including paired t-test, analysis of covariance (ANCOVA) and Pearson correlation coefficient were used. Data were analyzed using SPSS version 20. P values less than 0.05 were considered as significant.

## 4. Results

The mean age of elders in this study was 65.9 ± 3.6 year (elders aged 60 to 73 years old). Most of our study population elders were married (57.3%) and had high school education or below (75.2%). Also, 57.3% of elders (266 individuals) lived with their spouse and children. Regarding the question "how do you evaluate your health conditions at the moment?" most elders reported their health conditions as good (63.4%). Furthermore, 88.8% of individuals (206 persons) in intervention group and 87.5% (203 persons) in control group were insured. Also, 46.6% (108 individuals) in intervention group and 71.1% (165 individuals) in control group reported having a chronic disease. Given financial status, 83.6% (194 individuals) in intervention group and 82.8% (192 individuals) in control group earned their living through retirement salary, funds from other organizations and were employed (stable status). In addition, 54.7% (127 individuals) in intervention group and 51.3% (119 individuals) in control group owned their houses. Other featured of elders participating in the study based on intervention and control groups are shown in Table 3.

The results of paired t-test showed that mean scores difference of total healthy lifestyle index and its six dimensions including physical activity, nutrition, interpersonal relations, stress management, spiritual growth, and health responsibility and mean scores of total perceived social support index and its five dimensions including intimacy, assistance, social integration, affirmation of worth, and nurturance were significant in intervention group before and after the educational intervention (P value < 0.0001).

In the control group, the mean scores difference of healthy lifestyle and it’s six dimensions and the mean scores of social support and it’s five dimensions were significant before and after the educational intervention by using pair t test (P value < 0.0001) (Tables 4 and 5).

Results of analysis of covariance showed that by adjusting the effect of pre-test scores, the differences between the mean post test scores of healthy lifestyle and its six dimensions as well as perceived social support and its five dimensions in the control and intervention groups were statistically significant (P value < 0.0001) (Table 6).

In other words, it seems that health-promoting interventions have been effective in promoting dimensions and total scores of healthy lifestyle and perceived social support indexes in intervention group. In the double-variable analytical level, results of Pearson correlation coefficient test showed that total lifestyle index has a statistically significant and positive correlation with total perceived social support index based on intervention and control groups (P value < 0.05).

**Figure 2.**
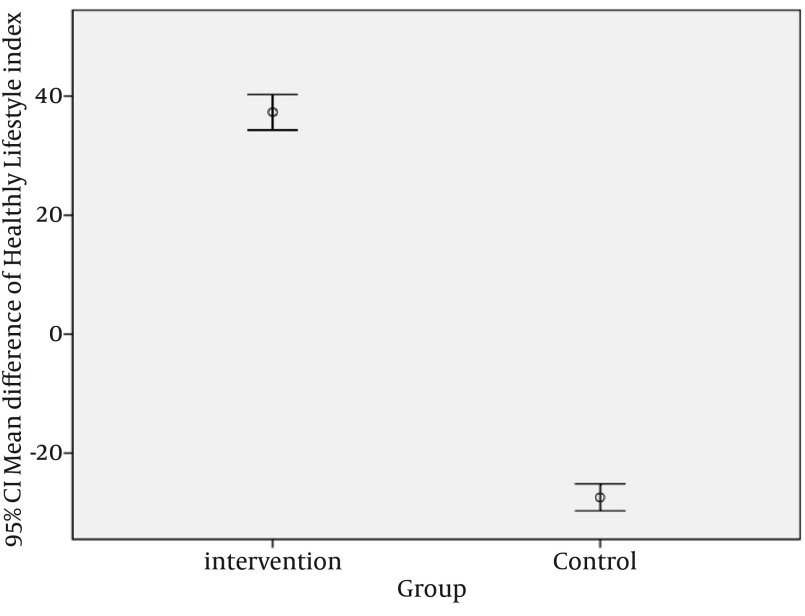
Comparing the Mean Difference of Total Healthy Lifestyle and Total Social Support Index Scores Between Intervention and Control Group

We compared the mean differences of total health promoting lifestyle and total social support scores between two groups. The mean difference of total health promoting lifestyle and total social support scores was calculated by mean total health promoting lifestyle and social support scores in pre intervention phase minus mean total health promoting lifestyle and total social support scores in post intervention phase, which was significantly greater in intervention than the control group. Independent samples t-test showed that there were significant differences in means of total health promoting lifestyle and total social support scores between the two groups (P < 0.0001). These results were presented by error bar plot in Figure 2.

**Table 3.** Characteristics of Elders in the Intervention and Control Group^a^

Variables	Intervention (n = 232)	Control (n = 232)	P Value
**Age**			0.64
> 65	122 (52.6)	117 (50.4)	
< 65	110 (47.4)	115 (49.6)	
**Sex**			0.78
Male	116 (50)	116 (50)	
Female	116 (50)	116 (50)	
**Education**			< 0.0001
Elementary	93 (40.1)	127 (54.7)	
Middle	58 (25)	71 (30.6)	
Higher	81 (34.9)	34 (14.7)	
**Marital status**			0.008
Married	148 (63.8)	118 (50.9)	
Widowed/Divorced	68 (29.3)	100 (43.1)	
Single	16 (6.9)	14 (6)	
**Housing status**			0.01
Landlord	127 (54.7)	119 (51.3)	
Tenant	105 (45.3)	113 (48.7)	
**Living structure**			< 0.0001
Family	177 (76.3)	148 (79.3)	
Relative	16 (6.9)	29 (12.5)	
Alone	39 (16/8)	19 (8.2)	
**Health status**			0.76
Good	144 (62.1)	150 (64.7)	
Average	65 (28)	58 (25)	
Bad	23 (9.9)	24 (10.3)	

^a^Data are presented as No. (%).

**Table 4.** Covariance Analysis of the Effect of Educational Intervention on Healthy Lifestyle and Its Six Dimensions, Perceived Social Support and Its Five Dimensions in the Intervention and Control Group

Indicators	df	Mean Square	F	P Value
**Lifestyle (Total)**				
Pre test	1	1107.57	4.63	0.03
Group	1	456786.50	1908	< 0.0001
**Physical activity**				
Pre test	1	16.13	1.68	0.20
Group	1	9941.20	1036	< 0.0001
**Nutrition**				
Pre test	1	2.84	0.17	0.68
Group	1	155558.81	912.99	< 0.0001
**Interpersonal relations**				
Pre test	1	14.55	0.78	0.38
Group	1	15801.43	848.4	< 0.0001
**Stress management**				
Pre test	1	38.33	3.84	0.05
Group	1	9916.82	994.09	< 0.0001
**Spiritual growth**				
Pre Test	1	97.69	8.30	0.004
Group	1	12521.54	1064	< 0.0001
**Health responsibility**				
Pre test	1	84.30	5.91	0.02
Group	1	13704.83	960.25	< 0.0001
**Social support (Total)**				
Pre test	1	2830.86	6.61	0.01
Group	1	278793.21	651.20	< 0.0001
**Intimacy**				
Pre test	1	50.72	0.56	0.45
Group	1	11465.77	126.77	< 0.0001
**Assistance**				
Pre test	1	12712.91	570.14	< 0.0001
Group	1	361.57	16.22	< 0.0001
**Social integration**				
Pre test	1	1.76	0.05	0.82
Group	1	11461.77	319.03	< 0.0001
**Affirmation of worth**				
Pre test	1	579.60	23.44	< 0.0001
Group	1	9730.64	393.48	< 0.0001
**Nurturance**				
Pre test	1	12009.65	5.68	0.02
Group	1	190.65	357.79	< 0.0001

**Table 5.** Mean Diffrences of Lifestyle Variables Before and After Hrealth Promoting Intervention^a,b^

Variables	Before Intervention	After Intervention	P Value
**Lifestyle (Total)**			
Intervention group (n = 232)	132.2 ± 19.7	169.5 ± 13.5	< 0.0001
Control groups (n = 232)	134.1 ± 8.1	106.7 ± 17.31	< 0.0001
**Physical activity**			
Intervention group (n = 232)	20.3 ± 3.9	25.6 ± 3	< 0.0001
Control groups (n = 232)	20.6 ± 2.3	16.3 ± 3.3	< 0.0001
**Nutrition**			
Intervention group (n = 232)	23.8 ± 3.9	29.7 ± 4.6	< 0.0001
Control groups (n = 232)	23 ± 2.5	18.1 ± 3.5	< 0.0001
**Interpersonal relations**			
Intervention group (n = 232)	22.8 ± 3.7	30.1 ± 4.8	< 0.0001
Control groups (n = 232)	23.1 ± 2.5	18.4 ± 3.8	< 0.0001
**Stress management**			
Intervention group (n = 232)	21 ± 4.01	25.9 ± 2.8	< 0.0001
Control groups (n = 232)	21 ± 2.3	16.7 ± 3.5	< 0.0001
**Spiritual growth**			
Intervention group (n = 232)	22.7 ± 3.6	28.9 ± 3.2	< 0.0001
Control groups (n = 232)	23.25 ± 2.4	18.6 ± 3.7	< 0.0001
**Health responsibility**			
Intervention group (n = 232)	23 ± 4	29.2 ± 4.05	< 0.0001
Control groups (n = 232)	23.1 ± 2.3	18.4 ± 3.5	< 0.0001

^a^Data are presented as Mean ± SD.

^b^P Values are significant.

**Table 6.** Mean Differences of Social Support Variables Before and After Health Promoting Intervention^a,b^

Variables	Before Intervention	After Intervention	P Value
**Social support (Total)**			
Intervention group (n = 232)	106.56 ± 22.7	144.20 ± 17.9	< 0.0001
Control groups (n = 232)	118.85 ± 13.7	94.21 ± 23.4	< 0.0001
**Intimacy**			
Intervention group (n = 232)	20.56 ± 6	29.5 ± 12. 02	< 0.0001
Control groups (n = 232)	24.49 ± 4.3	19.12 ± 6	< 0.0001
**Assistance**			
Intervention group (n = 232)	21.16 ± 5.1	28.34 ± 3.7	< 0.001
Control groups (n = 232)	22.61 ± 3.5	18.02 ± 5.7	< 0.0001
**Social integration**			
Intervention group (n = 232)	21.23 ± 5.9	29 ± 5.8	< 0.0001
Control groups (n = 232)	23.78 ± 4.2	18.71 ± 6.1	< 0.0001
**Affirmation of worth**			
Intervention group (n = 232)	21.02 ± 4.2	27.95 ± 3.9	< 0.0001
Control groups (n = 232)	23.54 ± 3.7	19.05.5 ± 6.1	< 0.0001
**Nurturance**			
Intervention group (n = 232)	22.58 ± 5.5	29.41 ± 5.9	< 0.0001
Control groups (n = 232)	24.42 ± 4.15	19.30 ± 5.8	< 0.0001

^a^Data are presented as Mean ± SD.

^b^P Values are significant.

## 5. Discussion

The hypothesis of this study was that health-promoting interventions lead to changes in the average scores of healthy lifestyle index and perceived social support. Results of statistical tests showed a significant statistical difference in total average scores of lifestyle between intervention and control groups after education. This significant difference was observed in all dimensions of lifestyle, while no changes were observed in control group. This indicates the positive effect of health-promoting interventions on elders' lifestyle and improvement of conditions of participants in intervention group. This result conforms to the results of the study conducted by Robinson-Whelen (17) and Saffari et al. (18), concluding that If elders carry out health-promoting behaviors regularly and correctly, they will have a healthy lifestyle (11). Many problems in old age originate from unhealthy lifestyle such as unhealthy nutrition and little physical activity. In old age, energy is less required but the need for nutrition does not change. Thus, keeping a diet and nutritional considerations are vital for elders. Immobility leads to many physical diseases, loneliness, and depression, which disables the person in doing daily activities and makes them dependent on others. A 30-minute simple and standard walking every day in 10-minute intervals enables elders to achieve balance and harmony, increase their muscular strength and flexibility and reduce their stress (19, 20). The best way to prevent depression, which is typical of this age is continuing social activities (12). Also, stress management is essential for maintaining and promoting long term healthy behavior patterns (7). It is impossible to delete stress but it is possible to learn how to overcome it. Identifying stimulants, pursing a relaxing activity like yoga, meditation, breathing slowly and deeply, doing regular exercises, listening to music, and reflective journaling are different ways to manage stress (7). Moreover, maintaining the communications with others especially relatives, children, and grandchildren is effective in stress management (21). Using stress management techniques in the current study promoted mental health and led to the better control over stress in intervention group as compared with control group. This was in line with the results of Dale et al. study (22). Sufficient sleep is a major component of health in old age and current study considers it as well. Elders, due to aging have numerous problems with sleeping (23). Determining regular hours for sleeping, having a daily program for walking, eating light meals, and drinking warm milk can promote the quality and quantity of sleeping (24). One of the other frequent complaints of the elders is memory deterioration, which unintentionally and unknowingly influences their lifestyles and makes them behave in a way that puts their health in jeopardy (25). The more the mental powers are used, the healthier the person is. Elders should try to stay mentally active and establish contacts with relatives, friends, and neighbors (7). Keeping a diary, recounting past memories, looking at photo albums, watching past movies, and listening to music are among interventions that improve elders' memories and delay Alzheimer in them to a certain degree (4). Individuals are inclined to change their behaviors when they consider their health worthy; in lifestyle, this is referred to as responsibility for health. Responsibility for health means acting wisely when health is threatened and this becomes more important in old age because not acting when health is threatened can have irreparable effects in these ages and even can cause the early death of the elder (26).

Elders who are mentally in a more optimal situation have more motivations to carry out health-promoting behaviors (27). This mental health is reinforced by being with family and having intimate relations with its members and effects elder's spiritual health. Studies show that elders who live at home with their families are in better conditions and have a more enjoyable life (28). Social dignity is considered to be one of the most important elements of elders' spiritual health. When they feel useful, their lives are more meaningful to them (29).

Elders who have the benefit of social support have a higher quality of life and are less vulnerable if they experience unfavorable incidents (30). Staying connected especially having significant communications, which are high in quality is a fixed predictor for the quality of life. Not being connected to others leads to isolation, depression, and seclusion and increases death rate by 3-7 times (5). Elders receive less social support because they do not attend social situations often due to their age and suffering from different disorders (26). However, social support perception is more important than its reception (8). When people realize that they can count on other people's help when need arises, they do not feel lonely and will ask for help. The present article focused on perceived social support. Results of statistical tests showed a positive effect of health-promoting interventions on the score of perceived social support in elders and reveals better conditions of intervention group, which is in line with other studies (20, 31). Having an intimate relation with a person to whom elders can express their feelings is of crucial importance (4). Individuals need to be in contact with others and be part of a group, which help elders to enhance their dignity and honor and ensure that there is source to which they can refer when they are in trouble.

The motto of World Health Organization for elders' day in 2013 was "what elders say". Elders usually seek respect and want to be considered and paid attention to. If respected by others, they feel satisfied, robust, and secure and find a context for more attempts and activities. Respecting elders can preserve their dignity and encourage them to do things to make changes in their lifestyle and promote its quality despite all difficulties (26).

Changing healthy behaviors are extremely difficult at individual level. If individuals have social support, they are more likely to succeed in changing their behaviors (32). The role of elders themselves is very important because it is important that they accept that they are old (29). Among interventions for accepting old age, one can refer to growing plants and flowers. These interventions keep elders engaged and increase their self-confidence.

It is impossible to prevent aging, but it is possible to prevent "worse aging". As the present study showed health-promoting intervention along with perceived social support increase the possibility of picking out health-promoting behaviors and heading toward healthy lifestyle in this period. This study recommends that educating healthy lifestyle promoting behaviors start before old age, while the person is middle-aged and even when they are in their teens or childhood.

The strength of this study is the design of randomized controlled trial and the weakness is the focus of study on elderly people who were admitted at health houses of municipality districts and lack of attention to others in nursing homes. Another limitation of this study is its focus on elders and their social-demographics characteristics while it overlooks the role of families, environment, laws, and policies.

## References

[1] Tajvar M, Arab M, Montazeri A (2008). Determinants of health-related quality of life in elderly in Tehran, Iran.. BMC Public Health..

[2] Lara J, Godfrey A, Evans E, Heaven B, Brown LJ, Barron E (2013). Towards measurement of the Healthy Ageing Phenotype in lifestyle-based intervention studies.. Maturitas..

[3] Kardakis T, Weinehall L, Jerden L, Nystrom ME, Johansson H (2014). Lifestyle interventions in primary health care: professional and organizational challenges.. Eur J Public Health..

[4] Sodergren M (2013). Lifestyle predictors of healthy ageing in men.. Maturitas..

[5] Halloran L (2012). Healthy Aging: Clinical and Lifestyle Considerations.. J Nurse Pract..

[6] Chakravarty EF, Hubert HB, Krishnan E, Bruce BB, Lingala VB, Fries JF (2012). Lifestyle risk factors predict disability and death in healthy aging adults.. Am J Med..

[7] Fiocco AJ, Scarcello S, Marzolini S, Chan A, Oh P, Proulx G (2013). The effects of an exercise and lifestyle intervention program on cardiovascular, metabolic factors and cognitive performance in middle-aged adults with type II diabetes: a pilot study.. Can J Diabetes..

[8] Centis E, Trento M, Dei Cas A, Pontiroli AE, De Feo P, Bruno A (2014). Stage of change and motivation to healthy diet and habitual physical activity in type 2 diabetes.. Acta Diabetol..

[9] Cao WJ, Chen CS, Hua Y, Li YM, Xu YY, Hua QZ (2012). Factor analysis of a health-promoting lifestyle profile (HPLP): application to older adults in Mainland China.. Arch Gerontol Geriatr..

[10] Baheiraei A, Mirghafourvand M, Mohammadi E, Charandabi SA, Nedjat S (2012). Social support for women of reproductive age and its predictors: a population-based study.. BMC Women Health..

[11] Hodge AM, English DR, Giles GG, Flicker L (2013). Social connectedness and predictors of successful ageing.. Maturitas..

[12] Sok SR, Yun EK (2011). A comparison of physical health status, self-esteem, family support and health-promoting behaviours between aged living alone and living with family in Korea.. J Clin Nurs..

[13] Whatley AD, DiIorio CK, Yeager K (2010). Examining the relationships of depressive symptoms, stigma, social support and regimen-specific support on quality of life in adult patients with epilepsy.. Health Educ Res..

[14] Alipor A, Sahraeian M, Aliakbari M, Aghababaei M (2012). [The relationship between perceived social support and hardiness with mental health and disability status among women with Multiple Sclerosis].. Res Soc Psychol..

[15] Ay S, Yanikkerem E, Calim SI, Yazici M (2012). Health-promoting lifestyle behaviour for cancer prevention: a survey of Turkish university students.. Asian Pac J Cancer Prev..

[16] Baheiraei A, Mirghafourvand M, Mohammadi E, Nedjat S, Charandabi SMA, Rajabi F (2011). Health-promoting behaviors and social support of women of reproductive age, and strategies for advancing their health: Protocol for a mixed methods study.. BMC Pub Health..

[17] Robinson-Whelen S, Hughes RB, Taylor HB, Colvard M, Mastel-Smith B, Nosek MA (2006). Improving the health and health behaviors of women aging with physical disabilities: A peer-led health promotion program.. Womens Health Issues..

[18] Saffari M, Amini N, Eftekhar Ardebili H, Sanaeinasab H, Mahmoudi M, Piper CN (2013). Educational intervention on health related lifestyle changes among Iranian adolescents.. Iran J Public Health..

[19] Kaur J, Kaur G, Ho BK, Yao WK, Salleh M, Lim KH (2014). Predictors of Physical Inactivity Among Elderly Malaysians: Recommendations for Policy Planning.. Asia Pac J Public Health..

[20] Waites C, Kaiser A, Martin F (2014). Health Promotion for African American Elders: Church is the Likely Place.. Int Perspectives Aging..

[21] Soffer M (2010). The role of stress in the relationships between gender and health-promoting behaviours.. Scand J Caring Sci..

[22] Dale H, Brassington L, King K (2014). The impact of healthy lifestyle interventions on mental health and wellbeing: a systematic review.. Mental Health Review J..

[23] Sodergren M, Wang WC, Salmon J, Ball K, Crawford D, McNaughton SA (2014). Predicting healthy lifestyle patterns among retirement age older adults in the WELL study: a latent class analysis of sex differences.. Maturitas..

[24] Chamroonsawasdi K, Phoolphoklang S, Nanthamongkolchai S, Munsawaengsub C (2010). Factors Influencing Health Promoting Behaviors among the Elderly Under the Universal Coverage Program, Buriram Province, Thailand.. Asia J Pub Health..

[25] Sola A, Nikpour S, Seyedoshohadaei M, Haghani H (2009). Health Promoting Behaviors and its Related Factors in Elderly.. Iran J Nurse..

[26] Van Malderen L, Mets T, Gorus E (2013). Interventions to enhance the Quality of Life of older people in residential long-term care: A systematic review.. Ageing Res Reviews..

[27] Huang SL, Li RH, Tang FC (2010). Comparing disparities in the health-promoting lifestyles of Taiwanese workers in various occupations.. Ind Health..

[28] Beser A, Bahar Z, Buyukkaya D (2007). Health promoting behaviors and factors related to lifestyle among Turkish workers and occupational health nurses' responsibilities in their health promoting activities.. Ind Health..

[29] Horning SM, Davis HP, Stirrat M, Cornwell RE (2011). Atheistic, agnostic, and religious older adults on well-being and coping behaviors.. J Aging Study..

[30] Coey-Boerner KA (2010). The Relationship Between Social Support And Health-Promoting Lifestyle Behaviors In Uninsured Adults..

[31] Rana AM, Wahlin AK, Sta C, Lundborg L, Kabir ZN (2009). Impact of health education on health-related quality of life among elderly persons: results from a community-based intervention study in rural Bangladesh..

[32] Wang RH, Chen SW, Tang SM, Lee SL, Jian SY (2010). The relationship between selected developmental assets and health-promoting behaviours of adolescents in Southern Taiwan.. J Clin Nurs..

